# A Survey on Daily Activity Inclination and Health Complaints among Urban Youth in Malaysia

**DOI:** 10.1155/2020/9793425

**Published:** 2020-12-09

**Authors:** Ai−Hong Chen, Saiful Azlan Rosli, Jeffery K. Hovis

**Affiliations:** ^1^Optometry, Faculty of Health Sciences, Universiti Teknologi MARA (UiTM), Cawangan Selangor, Kampus Puncak Alam, 42300 Puncak Alam, Selangor, Malaysia; ^2^School of Optometry and Vision Science, University of Waterloo, 200 University Avenue West, Waterloo, ON, Canada

## Abstract

Environmental influence is one of the attributing factors for health status. Chronic interaction with electronic display technology and lack of outdoor activities might lead to health issues. Given the concerns about the digital impact on lifestyle and health challenges, we aimed to investigate the daily activity inclination and health complaints among the Malaysian youth. A self-administered questionnaire covering lifestyle and health challenges was completed by 220 youths aged between 16 and 25. There were a total of 22 questions. Seven questions inspected the patterns of indoor and outdoor activities. Fifteen questions focused on the visual and musculoskeletal symptoms linked to both mental and physical health. The total time spent indoors (15.0 ± 5.4 hours/day) was significantly higher than that spent outdoors (2.5 ± 2.6 hours/day) (*t* = 39.01, *p* < 0.05). Total time engrossed in sedentary activities (13.0 ± 4.5 hours/day) was significantly higher than that in nonsedentary activities (4.5 ± 3.8 hours/day) comprised of indoor sports and any outdoor engagements (*t* = 27.10, *p* < 0.05). The total time spent on electronic related activities (9.5 ± 3.7 hours/day) was were higher than time spent on printed materials (3.4 ± 1.6 hours/day) (*t* = 26.01, *p* < 0.05). The association of sedentary activities was positive in relation to tired eyes (*χ*^2^ = 17.58, *p* < 0.05), sensitivity to bright light (*χ*^2^ = 12.10, *p* < 0.05), and neck pain (*χ*^2^ = 17.27, *p* < 0.05) but negative in relation to lower back pain (*χ*^2^ = 8.81, *p* < 0.05). Our youth spent more time in building and engaged in sedentary activities, predominantly electronic usage. The health-related symptoms, both visual and musculoskeletal symptoms, displayed a positive association with a sedentary lifestyle and a negative association with in-building time.

## 1. Introduction

The industry revolution (IR) from the age of steam (IR 1.0: 1700s–mid-1800s), electricity (IR 2.0: mid-1800s–early 1900s), and computing (IR 3.0: ∼1970s) to this era emphasising personality and customization with artificial intelligence (IR 4.0) has resulted in many changes in our lifestyle [1]. This has made our lives completely different from that of our ancestors. Urbanisation is one of the effects of the industrial revolution [1]. There is transformation of human mobility pattern since IR 3.0 that influenced microlevel human behaviours and well-being and macrolevel social organization and change [2]. Urban living encourages sedentary lifestyles. Overpopulation, road traffic density, excessive use of motorized transportation, and too few public spaces have made the physical activity more difficult in cities [3]. The sedentary lifestyles and the absence of physical exercise are among the high-risk factors for mortality [4]. The combined effects of urbanisation (air pollution, sedentary lifestyles, and poor diet) contributed to the expanding worldwide epidemic of chronic diseases [5, 6]. Similarly, the number of hospital patients recorded, the patient from children and adults in Malaysia, increased by twofold over the past four years due to these changes in lifestyle [7]. The lack of social cohesion and safety issues associated with rapid urbanisation has limited outdoor activities and regular exercising and consequently irrefutable health benefits [8]. Most people spend more than 90 percent of their time in buildings [9]. Time allocated for exercise is also influenced by competing time demands and community features [10]. Most of them spend their time on outdoor activities only on weekends for recreation walking or other active activities such as cycling, swimming, gardening, or picnicking. Simple activities such as walking and running in urban could be a challenge to safety issues to a pedestrian, which involve a high volume of traffic and complexity of physical characteristics of the road space [11, 12]. Pedestrian has twice the risk of the walking injuries at the public parking area and seven times the risk at walkway in urban compared to the rural [11]. The public tend to avoid routine walking in urban areas compared to rural due to the health threats of road traffic accidents [13].

The invention of electricity and computers has changed working and leisure activities as compared to the previous era. Many activities have shifted from outdoor to indoor due to the replacement of natural daylight with artificial light as the light source. Increased exposure to electronic devices is likely to occur as these devices become more common at all levels of the educational system [14]. Younger generations will likely experience more electronic device exposures in their education system [14]. Chronic interaction with electronic display technology might lead to eye-related symptoms and other health concerns [15]. Malaysia was ranked top five globally and the highest in Southeast Asia for mobile social media penetration [16]. Internet usage in Malaysia was 80% with users spending a daily average of eight hours and five minutes online [16]. There has been a resurgence of visual ergonomics to address the complications associated with electronic devices dominating lifestyle in this digital era [17].

The changes of youth's connectedness to natural and artificial environment in the digital era and its impact on lifestyles have been reported [18–23]. The potential excessive engagement in electronic gadgets by youth might be a health concern. Chronic pain could be associated with both physical and mental health [24–27]. Given the concerns about the digital impact on lifestyle and health challenges, we aimed to investigate the daily activity inclination and health complaints among the youth in Malaysia.

## 2. Materials and Methods

This study adhered to the Declaration of Helsinki and was approved by the institutional ethics review board. Three hundred urban youths were approached using convenient sampling. Our target participants closely represented the youth of Malaysia because the composition of youth in the chosen university was comprised of youth from all thirteen states of Malaysia. The lifestyle variation among students from different universities in Malaysia was presumed to be trivial due to similarity in the topographical placement of most universities in township environments, macroclimates resemblance in all parts of Malaysia, and the standardization of academic requirement that needed to conform to Malaysia Quality Assurance. In order to be inclusive of the full age range of youth, additional respondents were recruited from a local secondary school. Approximately 73% (220 respondents) agreed to participate in the study. Informed consent was obtained from all respondents prior to their participation. The age range of 220 respondents was between 16 and 25 years and within the range of the youth category [28]. Our respondents (year of birth ranged from 1992 to 2001) grew up during the industrial revolutions 3.0 with exposure to electronic devices since childhood. All respondents were Malaysian. The mean and standard deviation for the age of the respondents was 20.3 ± 2.9 years. Approximately 88% (193) of the respondents were female. All the respondents had no known physical or intellectual abnormality.

**Figure 1:**
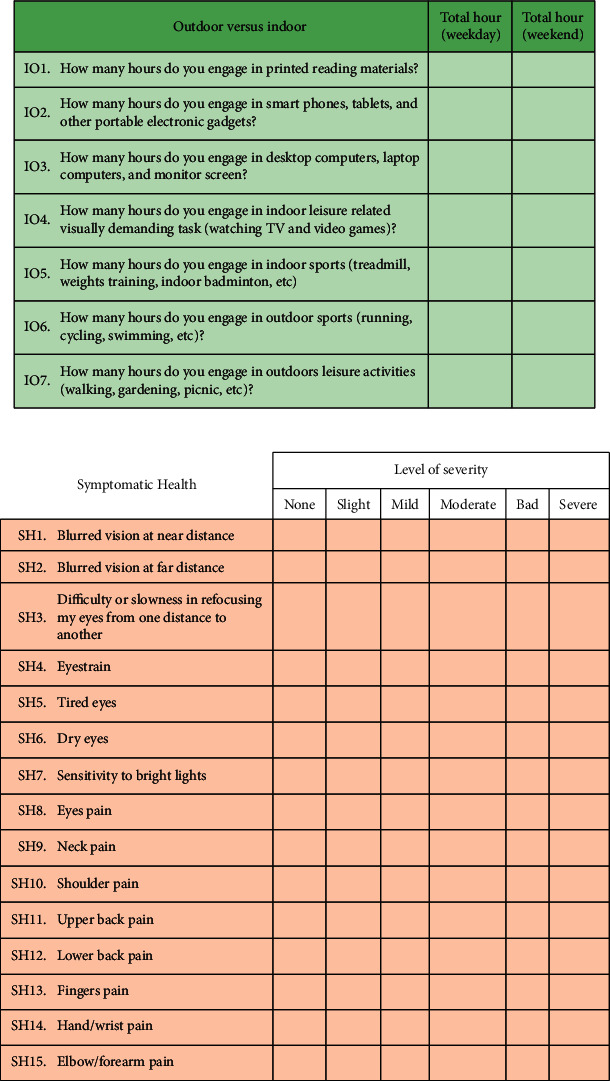
Lifestyle study in youth questionnaire (LSY). (a) Lifestyle in youth. (b) Health changes in youth (tick the level of severity of your visual and health issues).

Our Lifestyle Study in Youth (LSY) questionnaire was constructed based on the quality of life investigations [29, 30] and was self-administrated. The respondents were encouraged to ask the administrator for clarification if they were unsure of any terminology. Any ambiguity in terminology was clarified or explained to respondents in layman's terms by the administrator. The questionnaire and communication between the respondent and administrator were in English Language. Language proficiency was not an issue because English was taught as the second language in Malaysia. LSY, as shown in Figure 1, encompassed lifestyle investigations on outdoor-indoor activities as well as visual and musculoskeletal symptoms. There were in total 22 questions.

Lifestyle investigation examined how the respondents spent their time during the weekdays and weekends. Respondents entered their estimated hours spent on a specific activity on the weekday (Monday to Friday) and weekend (Saturday and Sunday). The average number of activity hours per day was calculated using the formula: (hours spent during a weekday *x* 5 + hours spent during a weekend day *x* 2)/7 [31]. Seven questions (IO1 to IO7) concerned the patterns of indoor and outdoor activities with three questions probing the usage of printed versus digital reading materials. Fifteen questions (SH1 to SH15) investigated the levels of visual and musculoskeletal symptoms. The severity of symptoms was rated on a 6-point scale (0, none; 1, slight; 2, mild; 3, moderate; 4, bad; and 5, severe). SH1 and SH2 addressed the severity of blurred vision from near and distance, respectively. SH3 examined the ability to shift focus from near to far and vice versa. SH4 to SH8 probed the five visual symptoms associated with near tasks [32]. These symptoms were eye strain, tired eyes, dry eyes, sensitivity to bright lights, and eye pain. SH9 to SH15 assessed the musculoskeletal discomfort associated with daily activities. Mental health state could exacerbate physical pain [24–27]. Chronic physical pain as indicated in SH9 to SH15 had been associated with mental health [24–27]. The questionnaire was included in the analysis only if all the questions were answered. Each respondent was given 30 minutes to complete the questionnaire.

## 3. Results

### 3.1. Lifestyle Pattern

The lifestyle of the youth was investigated from 4 aspects which are the time spent on indoor versus outdoor activities, sedentary preference analysis, electronic task engagement analysis, and the difference between weekdays and weekends.

### 3.2. Indoor and Outdoor Analysis

The lack of outdoor activities has been associated with many lifestyle diseases. The indoor-outdoor analysis helped us to understand the health risk of our youth. Seven questions examined the time spent indoors and outdoors and were used as an indicator of the youth's lifestyle.  Table 1 summarises the findings of these questions. The first 5 questions were categorised as indoor activities which encompassed daily activities of reading printed materials (IO1), reading electronic displays (IO2 and IO3), viewing electronic displays during indoor leisure activities (IO4), and participating in indoor sports (IO5). Two questions were categorised as outdoor activities. These were the time spent in outdoor leisure activities (IO6) and outdoor sports (IO7). The total time spent indoors (15.0 ± 5.4 hours per day) was found to be significantly higher than outdoors (2.5 ± 2.6 hours per day) (*t* = 39.01, *p* < 0.05). Our survey captured approximately 17.5 ± 7.1 hours per day of activities (both indoors and outdoors) among the youth. If we assumed that the remaining hours (6.5 hours/day) were used for sleeping and house chores, then it can be deduced that our youth spent approximately 21.5 ± 5.4 hours per day in buildings, which is about 90% of the total hours in one day.

### 3.3. Sedentary Analysis

Active lifestyle has been well acknowledged as a preventive measure for noncommunicable diseases. In terms of sedentary lifestyle analysis, we regrouped our findings to examine the level of physical activity either for work or leisure. Findings from questions IO1, IO2, IO3, and IO4 reflected activities that were sedentary in nature. Findings from IO5, IO6, and IO7 on indoor sports, outdoor leisure, and outdoor sports, respectively, were considered as nonsedentary activities. Our youth spent approximately 13.0 ± 4.6 hours per day in sedentary activities, significantly higher than nonsedentary activities (4.5 ± 3.8 hours per day) (*t* = 27.10, *p* < 0.05).

### 3.4. Electronic Task Engagement Analysis

Excessive engagement in electronic gadgets was associated with social isolation and health concerns. Findings from questions IO2, IO3, and IO4 were used to analyse the total time spent viewing electronic displays, and IO1 was used to determine the time spent viewing printed materials. Our youth spent approximately 9.5 ± 3.7 hours per day viewing electronic displays, which was significantly higher than the time spent viewing printed materials (3.4 ± 1.6 hours per day) (*t* = 26.01, *p* < 0.05).

### 3.5. Competing Time Analysis

Because the survey was taken while the institution was in session, one might expect the behaviour patterns to be different on the weekend since there are potentially fewer structured activities indoors. Although there was a difference in the time spent indoors, the youth spent significantly more time indoors during the weekend (16.5 ± 9.5 hours per day) as compared to the weekdays (14.3 ± 5.5 hours per day) (*t* = 3.60, *p* < 0.05). The total time spent outdoors per day was similar between the weekend (2.5 ± 3.2 hours per day) and the weekdays (2.5 ± 2.7 hours per day). The time allocated for the sedentary activities was higher during the weekend (14.6 ± 8.1 hours per day) as compared to the weekdays (12.3 ± 4.7 hours per day) (*t* = 4.25, *p* < 0.05).

### 3.6. Health Issues in Youth

Table 2 summarises the responses to the 15 questions on visual and musculoskeletal symptoms. For analysis, the responses were pooled into two categories: negligible category (none-to-mild rating) and symptomatic category (moderate-to-severe rating). Visual and musculoskeletal symptoms were found to be very common among the youth. More than half of the respondents reported tired eyes (67%), sensitivity to bright lights (64%), and blurred vision from far (56%). For the seven musculoskeletal symptoms listed (SH9 to SH15), most of the respondents experienced moderate-to-severe pain at the neck (67%), shoulder (68%), upper back (67%), and lower back (70%).

### 3.7. Interaction between Lifestyle Patterns to Predict the Symptom

Logistic regression was performed to determine whether symptoms could be predicted from their hourly spent indoor and outdoor, sedentary, and nonsedentary, and electronic- and nonelectronic-related activities. Preliminary tests on multicollinearity between the independent variables (hours spent on indoor, sedentary, and electronic activities) showed that the variance inflation factors (VIF) were less than 10, which indicated that multicollinearity is not a serious concern [33, 34]. It was hypothesized that more hourly spent indoor, sedentary, and electronic-related activities would be the major factors associated with moderate-to-severe symptoms. This analysis was performed using symptoms as the dependent variable. The youth were categorised into the nonsymptomatic group if the youth has negligible to mild symptoms, or categorised into the symptomatic group for moderate to severe symptoms. Referring to the highlighted column in Table 2, only seven symptoms had more than 50% of the youth reporting moderate-to-severe symptoms, as in Table 3.

There were two distinct patterns of interaction for the remaining five symptoms as indicated by the odds ratios (OR). The OR of more than one denotes a positive association (OR > 1), where the increasing hourly spend indoor, sedentary, and electronic related activities are associated with an increased likelihood of exhibiting symptoms. An OR of less than one signifies a negative association (OR < 1), where the increasing hourly spent indoor, sedentary, and electronic-related activities were associated with a reduction in the likelihood of exhibiting symptoms [35]. Positive associations occurred between tired eyes, sensitivity to bright light, neck pain, and lower back pain towards the predictor of hourly spent sedentary activities. Only lower back pain symptoms had positive associations towards the predictor of hourly spent electronic related activities. Meanwhile, negative associations (OR < 1) occurred between tired eyes, sensitivity to bright light, neck pain, and lower back pain towards the predictor of hours spent indoors. The lower back pain symptom has negative associations towards the predictor of hourly spent sedentary activities.

## 4. Discussion

### 4.1. Lifestyles

Time spent indoors was significantly higher than time spent outdoors in our study. Our youth displayed similar high percentages of time in buildings (89.58%) compared with the previous 90% values reported in other studies which included sleep time [9, 36]. Our youth spent about 15 hours/day on indoor activities. This value is higher than the 10.4–13.2 hours/day reported in developed countries [37]. The youth spent more time in indoor sedentary activities (watching TV and video games) than nonsedentary activities such as indoor sports. This result would be expected during weekdays because the institution was in session during the time of the survey, but our results showed that there was actually an increase in the time spent on sedentary activities during weekends. The Malaysian government launched a healthy lifestyle campaign in 1991 to improve the knowledge and the practices of healthy lifestyles among Malaysians [38]. Based on previous results for Malaysian youth [39], we assume that the youth in this survey had adequate knowledge about healthy lifestyles; however, our results indicated that this knowledge has not been translated into practice. We are uncertain whether the sedentary lifestyle is a result of urbanisation or ubiquitous electronic displays or a combination of both. If electronic devices are playing a major role, then the urban planning to encourage an active lifestyle may not be sufficient.

Approximately 40% of a day was spent on electronic device-related activities. Electronic display activities either for leisure (watching television, video games, and Internet use) or for work (computer use for work purposes) contributed to the increment of sedentary time. In developed countries, the usage of electronic devices was almost similar as in our study which was approximately 10−12 hours/day from stationary display devices (personal computer) to mobile display devices like smartphone, tablet, or laptop [40]. Although tablet or laptop usages have not completely replaced the printed media in our institution system, approximately 99% of our youth are engaged with portable electronic devices on weekdays, which was 95.4% higher than that reported twelve years ago [41].

Our findings did not seem to support the competing time theory because the higher indoor preference and sedentary lifestyle during weekends indicated that this tendency was probably not for work or institution related tasks. On weekdays, our youth spent more time on academic-related tasks. At the weekend, the youth usually had personal time for hobbies, friends, or family. Our youth spent an extra 2 hours per day for indoor and sedentary activities and an extra 3 hours on electronic displays, mostly smaller portable devices such as the cell phone during weekends. A previous study that measured the logged daily screen time also showed an increase of 2 hours in viewing electronic displays on weekends as compared to weekdays [42]. These findings indicated that the digital lifestyle is largely embedded in youth weekly activities, which raised health concerns. Despite the availability of green park and recreation amenities in an urban area, the stigma of safety especially on pedestrian hazard remained a concern in the public [11]. The traffic volume (i.e., hourly rate of cars), vehicular speed, and physical barriers between cars and pedestrians are likely to be the first public consideration to engage in outdoor activities [43]. Appropriate strategies not only enhance public safety but also encourage outdoor activities that help to improve both physical and mental health [44].

### 4.2. Health Issues

Health-related symptoms such as tired eyes, sensitivity to bright light, and neck pain were positively associated with a sedentary lifestyle, but not with the time spent indoors. Time spent indoor was grouped as multiactivity, which defined the activity related to being engaged with printed, electronic, and indoor physical activities. The previous finding showed that the eye symptoms were associated with the indoor environment, where the definition of indoor differed from our study [45]. This finding emphasised that the indoor parameter that causes the symptoms was usually due to humidity and temperature in a confined indoor space, which is out of our study scope. Lack of daily physical activities in the modern lifestyle predisposes the public to chronic diseases such as coronary artery diseases, hypertension, obesity, and diabetes mellitus [46]. Inactive physical activities give an impact on both physical and mental health [25–27, 47]. More hours spent on sedentary activities lead to health complaints such as tired eyes, sensitivity to bright light, and neck pain. Sedentary activities involve very low energy expenditure such as reclining, seated, or lying position [48]. Low energy expenditure usually relates to overweight and obesity, which gives high-risk factors for mortality [4]. Sedentary activities involving electronic tasks may indicate an ergonomic issue linked to visual and neck pain problems [49]. The long-term health risks of a sedentary lifestyle are a major concern. Inactive physical activity is positively associated with a mental health problem such as depression/anxiety disorder [25–27, 50]. Similar visual symptoms such as tired eyes and sensitivity to bright light have a strong association to depression and anxiety, which can be linked to changes of tear serotonin concentration linked to depression and anxiety disorders [51]. The bidirectional association between physical activities and brain function reduces neurological health conditions, such as Parkinson's disease, Alzheimer's disease, depression, and cognitive functions [52]. Lower physical activity has also been associated with eye diseases such as glaucoma [53]. Visual and musculoskeletal symptoms signify potential future vision and health problems. An active lifestyle might be an alternative as a low-cost, noninvasive preventive measure.

Those who spend more time on electronic related task were more likely to have lower back pain. However, the time spent on electronic related tasks did not significantly predict blur from distance, tired eyes, sensitivity to bright light, neck pain, shoulder pain, and upper back pain. This finding differed from the previous study that computer or electronic usage was associated with asthenopia symptoms and musculoskeletal symptoms [15]. However, our study only showed a connection between electronic related activities and lower back pain. This result was consistent with another study carried out on respondents aged 18–25 years which showed that lower back pain was highly reported among those with heavy electronic usage, especially laptops in relation to low-height table posture [54]. The symptoms related to sedentary activities showed that our youth was susceptible to near work-induced stress. Harmon's theory suggested that the reduction in the working distance and improper lighting to work surfaces led to poor posture habits which could affect a visual problem [55]. Near work-induced visual problems included asthenopia, blurring vision, and dry eyes. In order to maintain clear vision from near, the eyes need to constantly work hard to sustain the accommodation by shifting the retinal location to conjugate to the near object location via increasing the crystalline lens dioptre power [56]. The youth usually have enough accommodation reserves to cope with this visual stress but the coping mechanism might deteriorate with age. Therefore, the manifestation of the symptoms might be more apparent at an older age. The youth seem to embark on the agility from the age factor to adapt or cope with postural induced physiological stress by frequent usage of the smartphone or small electronic devices [57]. Based on the Skeffington near point stress model, human physiology is incompatible with near work demands, which induces stress that responded by the convergence and accommodation [58]. Countermeasure strategies to overcome the stress included adaptation through convergence and accommodation mechanism or avoidance of the stress source [59]. Most tasks that relate to electronic performed were short but high in frequency. When the stress could not be compensated, the performance of the visual function could be compromised by the breakdown of schemata [60]. We hypothesized that the cumulative time spent at close working distance might post a visual health risk in the long term, which could be beneficial for further study. The cumulative effect of near work in sedentary or electronic related habits could be manifested to other problems such as obesity or refractive error, which is a major problem among the Malaysian and other country youth these days [61, 62].

Our world is being gravely affected by the pandemic of COVID-19 now with devastating health, economic, and social disruption. What is the impact on our main findings through the perceptiveness of this global virus pandemic? With the lockdown enforcement and mandatory social-distancing practice in certain countries, surging of a sedentary lifestyle and more electronic engagement are unavoidable due to the sudden switch to online alternatives in education, shopping, and meetings in coping with the pandemic of COVID-19. Nevertheless, greater awareness of a healthy lifestyle and the key role of public health are among the constructive elements that arise from this pandemic. Our findings advocate that reduction in sedentary activities and electronic engagement can be beneficial to reduce the health complaint. Changing our living space might be a way to achieve that. There are two interesting recommendations in a recent article published in relation to living space challenges for healthy, safe, and sustainable housing [63]. One of their investigated areas was about visible and accessible green elements and space. Another investigated area was about flexibility, adaptability, usability, and accessibility of indoor space. They suggested emphasising the presence of balconies or terraces, view of greenery from windows, and compatibility between different functions in future building constructions. Sedentary lifestyle and excessive electronic engagement might be reduced without going outdoors with such innovative built-in likes open space and greenery view Health education or awareness program can be embedded to educate proper electronic usage and healthy lifestyle among youth. If reduction of digital exposure is not possible due to work or study requirements, professional ergonomic adjustment can be a good alternative to optimize viewing position, working distance, size of task, luminance, and so on. Future research on the physical properties of electronic devices and the impact of excessive exposure on both physical and mental health can be explored further. It is imperative because data pertaining to the impact on human from such unprecedented intense exposure to electronic devices is lacking. Cumulative time spent on electronic devices can also be investigated if it poses health risks in long term [61, 62]. To promote a healthy lifestyle to contemporary cities and modern societies, the concept of “Healthy and Salutogenic City” can be adapted to emphasise the connection between morphological and functional features of urban context and public health [64].

## 5. Conclusions

Our youth spent more time in buildings and engaged in sedentary activities predominantly electronic usage. The health-related symptoms, both visual and musculoskeletal symptoms, displayed a positive association with sedentary lifestyle, but a negative association with in-building time. Therefore, we can conclude that it is the types of activities and not in-building time that posed high risks to health issues among the youth. Future research should focus on the short-term and long-term impact of electronic devices on youth. Health risk encompasses both physical and mental health. Experimental design to address the concern is preferable to provide more insightful details on the impact. Lockdown and social distancing in the event of any future global virus pandemic may aggravate similar health threats. Therefore, future research embracing multidisciplinary effort is necessary to prepare the community to maintain healthy and sustainable lifestyle.

## Figures and Tables

**Table 1:** Findings on lifestyle activities in youth.

LSY items	Mean total hours per day for weekday (*A*)	Mean total hours per day for weekend (*B*)	Mean total hours per day *C* = (*A* + *B*)/2
IO1. How many hours do you engage in printed reading materials?	3.5 ± 1.9	3.3 ± 2.3	3.4 ± 1.6
IO2. How many hours do you engage in smartphones, tablets, and other portable electronic gadgets?	4.5 ± 2.5	6.2 ± 4.0	5.0 ± 2.4
IO3. How many hours do you engage in desktop computers, laptop computers, and monitor screen?	3.6 ± 2.1	4.6 ± 3.2	3.9 ± 2.0
IO4. How many hours do you engage in indoor leisure-related visually demanding task (e.g., watching TV and video games)?	0.7 ± 1.3	0.7 ± 1.3	0.7 ± 1.3
IO5. How many hours do you engage in indoor sports (e.g., treadmill, weights training, and indoor badminton)?	2.0 ± 1.8	1.8 ± 2.2	2.0 ± 1.7
IO6. How many hours do you engage in outdoor sports (e.g., running, cycling, and swimming)?	1.3 ± 1.6	1.3 ± 1.7	1.3 ± 1.5
IO7. How many hours do you engage in outdoor leisure activities (e.g., walking, gardening, and picnic)?	1.1 ± 1.4	1.2 ± 1.6	1.2 ± 1.3

**Table 2:** Findings on symptomatic health pattern in youth.

LSY items	Total counts in each option (out of 220 respondents)						Pooled results	
	None	Slight	Mild	Moderate	Bad	Severe	Negligible (none to mild)	Symptomatic (moderate to severe)
SH1. Blurred vision at near distance	141 (64%)	33 (15%)	15 (7%)	22 (10%)	9 (4%)	0 (0%)	189 (86%)	31 (14%)
*SH2. Blurred vision at far distance*	*32 (15%)*	*40 (18%)*	*25 (11%)*	*62 (28%)*	*38 (17%)*	*23 (10%)*	*97 (44%)*	*123 (56%)*
SH3. Difficulty or slowness in refocusing my eyes from one distance to another	62 (28%)	82 (37%)	22 (10%)	39 (18%)	11 (5%)	4 (2%)	166 (75%)	54 (25%)
SH4. Eyestrain	45 (20%)	51 (23%)	59 (27%)	52 (24%)	12 (5%)	1 (1%)	155 (70%)	65 (30%)
*SH5. Tired eyes*	*13 (6%)*	*21 (10%)*	*38 (17%)*	*95 (43%)*	*50 (23%)*	*3 (1%)*	*72 (33%)*	*148 (67%)*
SH6. Dry eyes	47 (21%)	58 (26%)	66 (30%)	37 (17%)	11 (5%)	1 (1%)	171 (78%)	49 (22%)
*SH7. Sensitivity to bright lights*	*12 (5%)*	*20 (9%)*	*47 (21%)*	*93 (42%)*	*34 (15%)*	*13 (6%)*	*79 (36%)*	*141 (64%)*
SH8. Eyes pain	60 (27%)	55 (25%)	64 (29%)	32 (15%)	8 (4%)	1 (1%)	179 (81%)	41 (19%)
*SH9. Neck pain*	*16 (7%)*	*27 (12%)*	*30 (14%)*	*92 (42%)*	*45 (20%)*	*10 (5%)*	*73 (33%)*	*147 (67%)*
*SH10. Shoulder pain*	*14 (6%)*	*29 (13%)*	*28 (13%)*	*79 (36%)*	*62 (28%)*	*8 (4%)*	*71 (32%)*	*149 (68%)*
*SH11. Upper back pain*	*28 (13%)*	*21 (10%)*	*24 (11%)*	*88 (40%)*	*51 (23%)*	*8 (4%)*	*73 (33%)*	*147 (67%)*
*SH12. Lower back pain*	*28 (13%)*	*23 (10%)*	*14 (6%)*	*74 (34%)*	*74 (34%)*	*7 (3%)*	*65 (30%)*	*155 (70%)*
SH13. Fingers pain	108 (49%)	62 (28%)	20 (9%)	18 (8%)	11 (5%)	1 (1%)	190 (86%)	30 (14%)
SH14. Hand/wrist pain	82 (37%)	77 (35%)	27 (12%)	24 (11%)	7 (3%)	3 (1%)	186 (85%)	34 (15%)
SH15. Elbow/forearm pain	78 (35%)	73 (33%)	43 (20%)	19 (9%)	5 (2%)	2 (1%)	194 (88%)	26 (12%)

The columns in italics were the common symptoms reported (more than 50%).

**Table 3:** The *p* values for the association and Odds ratio with 95% confidence interval (CI) results for visual and musculoskeletal symptoms on indoor, sedentary, and electronic-related activities.

Symptomatic	Exposure	*B*	Se	Wald	D*f*	*p*	Odds ratio	95% CI for odds ratio		*χ* ^*2*^
	(Mean total hours per day)							Lower	Upper	
Blur at distance	Indoor	−0.04	0.09	0.25	1	0.62	0.96	0.8	1.14	1.86, 0.60
	Sedentary	0.15	0.13	1.17	1	0.28	1.16	0.89	1.5	
	Electronic	−0.08	0.11	0.52	1	0.47	0.92	0.74	1.15	
	Constant	−0.19	0.42	0.21	1	0.65	0.83			
Tired eye	*Indoor*	*−0.33*	*0.1*	*11.07*	*1*	*0.00* ^*∗*^	*0.72*	*0.59*	*0.87*	17.58, 0.00
	*Sedentary*	*0.51*	*0.15*	*11.43*	*1*	*0.00* ^*∗*^	*1.67*	*1.24*	*2.25*	
	Electronic	−0.23	0.13	3.38	1	0.07	0.8	0.62	1.02	
	Constant	1.22	0.45	7.28	1	0.01	3.37			
Sensitivity to bright lights	*Indoor*	*−0.3*	*0.1*	*9.93*	*1*	*0.00* ^*∗*^	*0.74*	*0.61*	*0.89*	12.10, 0.01
	*Sedentary*	*0.37*	*0.14*	*6.82*	*1*	*0.01* ^*∗*^	*1.45*	*1.1*	*1.92*	
	Electronic	−0.08	0.12	0.4	1	0.53	0.93	0.74	1.17	
	Constant	0.98	0.44	5.02	1	0.03	2.66			
Neck pain	*Indoor*	*−0.36*	*0.1*	*13.48*	*1*	*0.00* ^*∗*^	*0.7*	*0.57*	*0.84*	17.27, 0.00
	*Sedentary*	*0.41*	*0.15*	*7.79*	*1*	*0.01* ^*∗*^	*1.5*	*1.13*	*2*	
	Electronic	−0.05	0.12	0.2	1	0.66	0.95	0.75	1.2	
	Constant	1.4	0.46	9.4	1	0	4.03			
Shoulder pain	*Indoor*	*−0.27*	*0.1*	*8.05*	*1*	*0.01* ^*∗*^	*0.76*	*0.63*	*0.92*	13.10, 0.00
	Sedentary	0.23	0.14	2.71	1	0.1	1.26	0.96	1.66	
	Electronic	0.01	0.12	0.01	1	0.92	1.01	0.8	1.28	
	Constant	1.74	0.46	14.24	1	0	5.7			
Upper back pain	Indoor	−0.06	0.13	0.24	1	0.63	0.94	0.74	1.2	2.06, 0.56
	Sedentary	−0.1	0.19	0.28	1	0.6	0.91	0.63	1.31	
	Electronic	0.22	0.16	1.83	1	0.18	1.24	0.91	1.7	
	Constant	−1.58	0.59	7.3	1	0.01	0.21			
Lower back pain	Indoor	0.09	0.13	0.43	1	0.51	1.09	0.84	1.41	8.81, 0.03
	*Sedentary*	*−0.52*	*0.23*	*5.28*	*1*	*0.02* ^*∗*^	*0.6*	*0.38*	*0.93*	
	*Electronic*	*0.54*	*0.2*	*7.02*	*1*	*0.01* ^*∗*^	*1.71*	*1.15*	*2.54*	
	Constant	−1.8	0.68	7.1	1	0.01	0.17			

The columns in italics indicate statistical significance based on binomial logistic regression, ^*∗*^*p* < 0.05.

## Data Availability

The data used to support the findings of this study are available from the corresponding author upon request.
